# Brain microstructural properties related to subjective well-being: diffusion tensor imaging analysis

**DOI:** 10.1093/scan/nsab063

**Published:** 2021-05-14

**Authors:** Chiaki Terao Maeda, Hikaru Takeuchi, Rui Nouchi, Ryoichi Yokoyama, Yuka Kotozaki, Seishu Nakagawa, Atsushi Sekiguchi, Kunio Iizuka, Sugiko Hanawa, Tsuyoshi Araki, Carlos Makoto Miyauchi, Kohei Sakaki, Takayuki Nozawa, Shigeyuki Ikeda, Susumu Yokota, Daniele Magistro, Yuko Sassa, Yasuyuki Taki, Ryuta Kawashima

**Affiliations:** Department of Nuclear Medicine and Radiology, Institute of Development, Aging and Cancer, Tohoku University, Sendai, Miyagi 980-8575, Japan; Division of Developmental Cognitive Neuroscience, Institute of Development, Aging and Cancer, Tohoku University, Sendai, Miyagi 980-8575, Japan; Department of Cognitive Health Science, Institute of Development, Aging, and Cancer, Tohoku University, Sendai, Miyagi 980-8575, Japan; Smart Aging Research Center, Tohoku University, Sendai, Miyagi 980-8575, Japan; Suwa Red Cross Hospital, Suwa, Nagano 392-8510, Japan; Division of Clinical research, Medical-Industry Translational Research Center, Fukushima Medical University School of Medicine, Fukushima 960-1295, Japan; Department of Human Brain Science, Institute of Development, Aging and Cancer, Tohoku University, Sendai, Miyagi 980-8575, Japan; Division of Psychiatry, Tohoku Medical and Pharmaceutical University, Sendai, Miyagi 983-8512, Japan; Department of Behavioral Medicine National Institute of Mental Health, National Center of Neurology and Psychiatry, Kodaira, Tokyo 187-0031, Japan; Division of Medical Neuroimaging Analysis, Department of Community Medical Supports, Tohoku Medical Megabank Organization, Tohoku University, Sendai, Miyagi 980-8573, Japan; Department of Psychiatry, Tohoku University Graduate School of Medicine, Sendai, Miyagi 980-8575, Japan; Department of Human Brain Science, Institute of Development, Aging and Cancer, Tohoku University, Sendai, Miyagi 980-8575, Japan; ADVANTAGE Risk Management Co., Ltd, Tokyo 153-0051, Japan; Department of Advanced Brain Science, Institute of Development, Aging and Cancer, Tohoku University, Sendai, Miyagi 980-8575, Japan; Department of Advanced Brain Science, Institute of Development, Aging and Cancer, Tohoku University, Sendai, Miyagi 980-8575, Japan; Research Institute for the Earth Inclusive Sensing, Tokyo Institute of Technology, Meguro, Tokyo 152-8552, Japan; Department of Ubiquitous Sensing, Institute of Development, Aging and Cancer, Tohoku University, Sendai, Miyagi 980-8575, Japan; Faculty of Arts and Science, Kyushu University, Fukuoka 819-0395, Japan; Department of Sport Science, School of Science and Technology, Nottingham Trent University, Nottingham NG11 8NS, UK; Division of Developmental Cognitive Neuroscience, Institute of Development, Aging and Cancer, Tohoku University, Sendai, Miyagi 980-8575, Japan; Department of Nuclear Medicine and Radiology, Institute of Development, Aging and Cancer, Tohoku University, Sendai, Miyagi 980-8575, Japan; Division of Developmental Cognitive Neuroscience, Institute of Development, Aging and Cancer, Tohoku University, Sendai, Miyagi 980-8575, Japan; Division of Medical Neuroimaging Analysis, Department of Community Medical Supports, Tohoku Medical Megabank Organization, Tohoku University, Sendai, Miyagi 980-8573, Japan; Division of Developmental Cognitive Neuroscience, Institute of Development, Aging and Cancer, Tohoku University, Sendai, Miyagi 980-8575, Japan; Department of Human Brain Science, Institute of Development, Aging and Cancer, Tohoku University, Sendai, Miyagi 980-8575, Japan; Department of Advanced Brain Science, Institute of Development, Aging and Cancer, Tohoku University, Sendai, Miyagi 980-8575, Japan

**Keywords:** subjective well-being, mean diffusivity, diffusion tensor imaging, dopaminergic system, motivation

## Abstract

Although it is known that health is not merely the absence of disease, the positive aspects of mental health have been less comprehensively researched compared with its negative aspects. Subjective well-being (SWB) is one of the indicators of positive psychology, and high SWB is considered to benefit individuals in multiple ways. However, the neural mechanisms underlying individual differences in SWB remain unclear, particularly in terms of brain microstructural properties as detected by diffusion tensor imaging. The present study aimed to investigate the relationship between measurements of diffusion tensor imaging [mean diffusivity (MD) and fractional anisotropy] and the degree of SWB as measured using a questionnaire. Voxel-based analysis was used to investigate the association between MD and SWB scores in healthy young adults (age, 20.7 ± 1.8 years; 695 males and 514 females). Higher levels of SWB were found to be associated with lower MD in areas surrounding the right putamen, insula, globus pallidus, thalamus and caudate. These results indicated that individual SWB is associated with variability in brain microstructural properties.

## Introduction

Every person hopes to lead a healthy life. Health is defined by the World Health Organization (WHO) as a state of complete physical, mental and social well-being and not merely the absence of disease or infirmity. Although this statement defines health as more than a lack of illness, neuropsychological investigations conducted of positive mental health have been fewer than those of psychological disorders and diseases.

### Subjective well-being and its components

Subjective well-being (SWB) is an indicator of positive psychology as well as happiness, life satisfaction and positive social influence (Sell and Nagpal, 1992). SWB is a broad category of phenomena that includes people’s emotional responses, domain satisfactions and global judgments of life satisfaction (Diener *et al.*, 1999). SWB is considered to have pleasant and unpleasant affective features and a cognitive aspect of life satisfaction (Diener and Suh, 1997). Numerous previous studies have suggested that positive affect (PA) and negative affect (NA) are moderately inversely correlated; however, they are perceived as distinct dimensions rather than opposite ends of the same dimension (Headey *et al.*, 1984; Diener *et al.*, 1999; Dodge *et al.*, 2012). According to previous research regarding SWB among patients with psychiatric disorders and non-clinical participants, the correlation between PA and NA was relatively high in patients and low in non-clinical participants (Ono *et al.*, 1996). Because a decrease in NA does not necessarily promote an increase in PA, it appears to be important to consider PA and NA separately when investigating SWB.

### Association between SWB and dopaminergic system

Previous neuropsychological studies have indicated that the dopaminergic system, including striatal areas, contributes to SWB. For instance, antipsychotic medications reduce SWB in patients with psychotic disorders (de Haan *et al.*, 2000; Bressan *et al.*, 2002; Mizrahi *et al.*, 2007). These drugs occupy the dopamine D2-receptor, resulting in a reduction of dopaminergic neurotransmission and suppression of psychotic symptoms (Seeman, 2002). Furthermore, blocking of dopamine receptors is associated with reduced motivation and emotional experiences based on natural rewards—processes related to endogenous dopaminergic activity (Kapur *et al.*, 2005)—and could be associated with a reduction in SWB. Other studies have observed subjective happiness scores to decrease in healthy subjects with acute dopamine depletion by alpha-methyl paratyrosine (Fujita *et al.*, 2000; Verhoeff *et al.*, 2001).

### Previous neuroimaging studies of SWB

Previous structural magnetic resonance imaging (MRI) studies demonstrated an association between SWB and various brain regions. One study showed that subjective happiness was positively associated with the regional gray matter volume (rGMV) in the rostral anterior cingulate cortex (Matsunaga *et al.*, 2016), whereas another study showed that it was positively associated with the rGMV in the precuneus (Sato *et al.*, 2015). Another study showed that an individual’s life satisfaction was positively correlated with the rGMV in the parahippocampal gyrus and negatively correlated with the rGMV in the precuneus and ventromedial prefrontal cortex (Kong *et al.*, 2015). These discrepancies may be derived from the differences in factors that are associated with SWB depending on the participants’ culture and context in which they live or the relatively small sample size of each study. As for functional MRI, there are growing findings from resting-state MRI studies using self-report assessment of SWB, indicating importance of the default mode network and emotional and rewarding system (Luo *et al.*, 2014; Shi *et al.*, 2018). We believe that further studies from diverse areas should be conducted on this topic. Accordingly, there are accumulating neuroimaging studies of SWB; however, to the best of our knowledge, studies examining the association between SWB and brain microstructural properties as detected by diffusion tensor imaging (DTI) are scarce.

### DTI measures and individual differences

DTI is a MRI technique that exploits the differences in the molecular diffusion of water according to tissue architecture (Basser *et al.*, 1994), including the molecular diffusion rate, directional preference of diffusion, and axial and radial diffusivity (Basser *et al.*, 1994; Basser and Pierpaoli, 1996; Le Bihan *et al.*, 2001). There are two popular measures of DTI—fractional anisotropy (FA) and mean diffusivity (MD). FA, the most common index of DTI, is used to represent the motional anisotropy of water molecules, being sensitive to the presence and integrity of white matter (WM) (Assaf and Pasternak, 2008), whereas MD is a scalar measure of the directionally averaged diffusion magnitude and is related to brain tissue integrity (Pierpaoli *et al.*, 1996). Compared with FA, MD can be used to assess the microstructural properties of broader brain structures, including gray matter.

In recent years, differences in MD have been shown to underlie individual cognitive differences and brain pathology (Piras *et al.*, 2010; Laricchiuta *et al.*, 2014). In particular, MD in areas strongly related to the dopaminergic system, particularly subcortical areas including the basal ganglia (globus pallidus, putamen and caudate nucleus) and thalamus, is reportedly associated with several conditions related to differences or changes of the dopaminergic system (Takeuchi and Kawashima, 2018). In our previous study, MD in such areas was robustly associated with some mood states, temperaments and cognitive functions related to dopaminergic function; it was suggested that an overlap of these correlates involved a motivational component (Takeuchi *et al.*, 2015). Considering previous findings that the dopaminergic system contributes to SWB (de Haan *et al.*, 2000; Verhoeff *et al.*, 2001; Mizrahi *et al.*, 2007), we hypothesized that SWB might be related to MD in the regions associated with the dopaminergic system.

In the present study, we aimed to investigate the relationship between SWB and DTI measures and to test the hypothesis of the association between SWB and MD, particularly MD in the regions associated with the dopaminergic system, using data from a large sample of healthy young adults. Accordingly, we used the Japanese version of the Subjective Well-Being Inventory (SUBI) and investigated the relationship between SUBI scores and DTI measures (MD or FA) via voxel-based analysis. Further, we investigated the association between SWB and the motivational state as measured by the Profile of Mood States (POMS) because motivational component is considered to play an important role in the MD in the regions associated with the dopaminergic system.

## Methods

### Participants

Overall, 1209 healthy, right-handed individuals (695 males and 514 females) participated in the present study. This research was a part of our ongoing project to investigate associations among brain imaging results, cognitive function, aging, genetics and daily habits. Mean participant age was 20.7 years (s.d., 1.78; range: 18–27 years). All participants were university students, postgraduates or university graduates of <1 year. Each participant had normal vision and none had a history of neurological or psychiatric illness. Handedness was evaluated using the Edinburgh Handedness Inventory (Oldfield, 1971). Written informed consent was obtained from each participant for projects in which they participated. The procedures for all studies were approved by the Ethics Committee of Tohoku University.

### Psychological measurements

#### Subjective Well-Being Inventory.

SWB can be quantified using the WHO SUBI (Sell and Nagpal, 1992). SUBI is a self-report questionnaire used to comprehensively assess the degree of an individual’s physical, mental and social well-being based on their own experiences. This questionnaire enables the measurement of two types of SWB independently: PA, an index of psychological healthfulness, and NA, an index of poor psychological healthfulness. SUBI questionnaire comprises 40 items, each rated on a 3-point scale. The items are divided into 11 subscales: sense of satisfaction (F1), sense of achievement (F2), self-confidence (F3), sense of happiness (F4), support of close relatives (F5), social support (F6), family relationships (F7), sense of spiritual control (F8), sense of physical ill health (F9), dissatisfaction with social ties (F10) and sense of disappointment (F11). PA comprises 19 items included in subscales F1–F7, and NA comprises 21 items included in subscales F7–F11. Because extremely few of the study participants had a spouse or children, three questions regarding family relationships (F7) were excluded. Subsequently, 37 items (18 items for PA and 19 for NA) divided into 10 subscales (from F1 to F6 for PA and from F8 to F11 for NA) remained. Examples of the questions associated with PA are ‘Do you feel your life is interesting?’ and ‘Do you think that most of the members of your family feel closely attached to one another?’. Each item was answered using a 3-point scale as follows: 3 = ‘I strongly agree,’ 2 = ‘I somewhat agree’ and 1 = ‘I disagree.’ Examples of the questions associated with NA are ‘Do you feel your life is boring?’ and ‘Do you get easily upset if things don’t turn out as expected?’. Each item is answered using 3-point scale as follows: 1 = ‘I strongly agree,’ 2 = ‘I somewhat agree’ and 3 = ‘I disagree.’ For both PA and NA, higher scores on each item indicate a better state of well-being. The reliability and validity of this scale has previously been demonstrated (Tonan *et al.*, 1995); the internal consistency of PA and NA, as measured with Cronbach’s coefficient α, was >0.8 (Ono and Yoshimura, 2010), indicating that the questionnaire was extremely reliable. The correlation coefficients between the 12-Item General Health Questionnaire (Goldberg and Williams, 1988) and both PA and NA were *r* = −0.43 and *r* = −0.57, respectively (Ono and Yoshimura, 2010), suggesting that the questionnaire was valid. Additionally, the SUBI questionnaire has previously been used to investigate the SWB of patients (Noguchi *et al.*, 2006; Fujisawa *et al.*, 2010; Iwamoto *et al.*, 2011) and non-clinical participants (Kanai *et al.*, 2016; Sano *et al.*, 2018).

#### Raven’s Advanced Progressive Matrix.

The Raven’s Advanced Progressive Matrix (RAPM; Raven, 2000), a widely used measure of general intelligence (Melby-Lervåg and Hulme, 2013), was applied to examine the effects of general intelligence on brain structures to exclude the possibility that the significant correlation between MD and SUBI scores was owing to either an association between the SUBI scores and general intelligence or an association between MD and general intelligence.

#### Profile of Mood States.

POMS (McNair *et al.*, 1992), a measure widely used for the assessment of mood states, was used in the present study. The shortened Japanese version (Yokoyama, 2005) of POMS was adjusted to examine the effect of mood states in the week preceding the use of SUBI questionnaire for each participant. The questionnaire comprised six subscales—tension/anxiety, depression/dejection, anger/hostility, vigor/activity (V/A), fatigue/inertia and confusion/bewilderment—each consisting of five items. Data for these measures were collected from 1193 study participants (data from 16 participants were missing). The validity of this measure has previously been demonstrated (Yokoyama, 2005); the high reliability of this measure was confirmed in a previous study conducted in our laboratory that included data from the same participants (Takeuchi *et al.*, 2017).

We did not include scales of this measure as covariates in the whole brain imaging analyses because we assumed that SWB would be fundamentally shared by the components of mood states; therefore, these effects were not regressed in the present study. Our previous study investigated the association between POMS and MD (Takeuchi *et al.*, 2017); we adopted this measure in the present study to complement the discussion of the main results.

#### Behavioral data analysis.

The behavioral data were analyzed using the IBM SPSS Statistics 21.0 software package (IBM Corp.; Armonk, NY, USA). Differences between males and females in age and psychological measure scores were analyzed using Mann–Whitney U-tests. In each analysis, *P*-value of <0.05 was considered significant. The potential association between PA and NA was investigated using multiple regression analysis with age and sex as covariates; a result with a threshold of *P*-value of <0.05 was considered significant. Moreover, the associations between each SUBI score and other psychological measures were assessed using multiple regression analyses. In each analysis, the dependent variable was the score of PA or NA, and the independent variables comprised one of the scores of the POMS or RAPM subscales, age and sex. These multiple regression analyses of PA and NA were separately performed because correcting one with the other may be inappropriate owing to multicollinearity. In these analyses, results with a threshold *P*-value of <0.05 were considered significant after correcting for the false discovery rate (FDR) using the classical one-stage method (Benjamini and Hochberg, 2000).

#### Image acquisition and analysis.

All MRI data were acquired using a 3T Philips Achieva scanner (Philips Medical Systems, Best, Netherlands). Diffusion-weighted data were acquired using a spin-echo echo-planar imaging (EPI) sequence [repetition time (TR) = 10 293 ms, echo time (TE) = 55 ms, field of view (FOV) = 22.4 cm, 2 × 2 × 2 mm^3^ voxels, 60 slices, SENSE reduction factor = 2, number of acquisitions = 1] with an 8-channel head-coil. The diffusion weighting was isotropically distributed along 32 directions (*b*-value = 1000 s/mm^2^). Three images with no diffusion weighting (*b*-value = 0 s/mm^2^) were acquired using a spin-echo EPI sequence (TR = 10 293 ms, TE = 55 ms, FOV = 22.4 cm, 2 × 2 × 2 mm^3^ voxels, 60 slices). Acquisitions for phase correction and for signal stabilization were performed; however, they were not used as part of the reconstructed images. FA and MD maps were calculated from the collected images using a commercially available diffusion tensor analysis package (Philips Medical Systems, Best, Netherlands) on the MR console. These procedures involved corrections for motion and distortion caused by eddy currents. All calculations were performed using a previously described method (Le Bihan *et al.*, 2001).

#### Preprocessing of imaging data.

Preprocessing of the imaging data was performed using Statistical Parametric Mapping software (SPM8; Wellcome Department of Cognitive Neurology, London, UK) implemented in MATLAB (Mathworks, Natick, MA). We adopted a previously validated two-step new segmentation algorithm (Takeuchi *et al.*, 2013) for segmentation of diffusion images. Thereafter, using the diffeomorphic anatomical registration via exponentiated lie algebra (DARTEL) registration process implemented in SPM8, the raw MD map, FA map, GM segmentation map [gray matter concentration (GMC) map], WM segmentation map [white matter concentration (WMC) map] and cerebrospinal fluid (CSF) segmentation map [CSF concentration (CSFC) map] were normalized; the voxel size for all normalized MD (or FA) images and segmented images was 1.5 × 1.5 × 1.5 mm^3^. The template for the DARTEL procedure was created in our previous study, which was developed using participants in the same project (see Takeuchi *et al.*, 2013). Subsequently, from the normalized images of the MD maps, the areas that were least likely to be GM or WM in the averaged normalized GMC and WMC images (defined as ‘gray matter tissue probability + white matter tissue probability < 0.99’) were removed to exclude the strong effects of CSF on MD throughout the analyses. From the normalized images of the FA, the areas that were least likely to be WM (defined as ‘white matter tissue probability < 0.99’) were removed. These images were then smoothed (8 mm full-width at half maximum for MD) and used for second-level analyses. We used SPM8 rather than SPM12 for all preprocessing because all methods and parameters were optimized and validated using SPM8 in our previous study (Takeuchi *et al.*, 2013). For more details of these procedures, see the Supplementary material.

### Statistical analysis

#### Whole brain statistical analysis.

The statistical analyses of imaging data were performed using SPM8. We performed multiple regression analyses to assess the relationship between MD or FA and the SUBI scores of PA or NA. The analyses of PA and NA were conducted separately because these scores are regarded as not completely independent, but only partly overlapping, and the correction of both as part of a multiple regression analysis may be inappropriate. Moreover, due to the strength of association between PA and NA, these scores were not analyzed in one multiple regression to avoid multicollinearity. These analyses were performed with sex, age, RAPM score, total intracranial volume (TIV), and PA or NA scores as covariates. Using the masks created as described above, the analyses for MD were limited to the areas strongly likely to be GM or WM, and those for FA were limited to the areas strongly likely to be WM. Correction for multiple comparisons was performed using threshold-free cluster enhancement (TFCE) (Smith and Nichols, 2009) with randomized non-parametric permutation testing (5000 permutations) using the TFCE toolbox (http://dbm.neuro.uni-jena.de/tfce/). We applied a family-wise error-corrected threshold *P*-value of <0.05. We used SPM8 for statistical analyses for compatibility with the software used for permutation-based statistics and the homemade script used for the statistical analyses. The results should not have been affected by the version of SPM used.

#### Analyses of sex differences in the neural correlates of SUBI scores.

We analyzed MD and FA correlates with respect to sex differences because the SUBI scores showed significant differences related to sex in behavioral analyses. Detailed descriptions about the sex differences in the MD and FA correlates of the SUBI scores are presented in the Supplementary material.

## Results

### Behavioral data

**Fig. 1.**
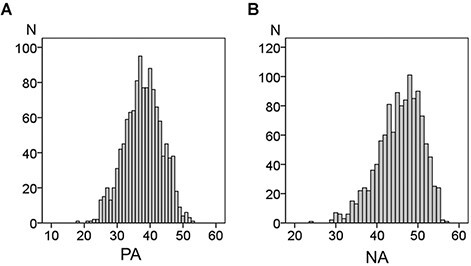
Distribution of SUBI scores. Histograms show the distribution of PA (A) or NA (B) scores.

**Fig. 2.**
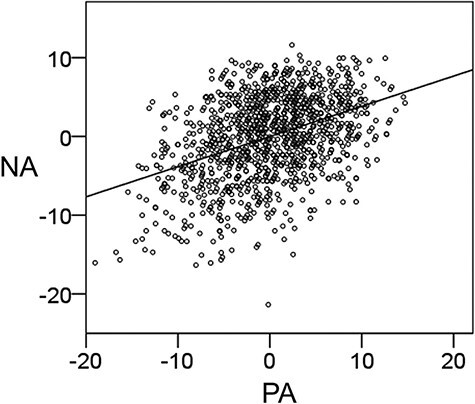
Association between PA and NA. Residual plots with trend lines depict the correlation between residuals of PA and NA in multiple regression analyses with covariates including age and sex.

Table 1 shows the mean values and ranges for age and SUBI-PA, SUBI-NA, RAPM and POMS scores in males and females. Mann–Whitney U-tests showed that females had a significantly higher SUBI score of PA, whereas males had a significantly higher score of NA (i.e. a higher level of SWB). Furthermore, there were significant differences between males and females in RAPM score and on the subscales of tension/anxiety, depression/dejection and anger/hostility of POMS. The multiple regression analysis for the association between PA and NA with covariates of age and sex revealed that there was a significant positive correlation (β = 0.410, *t* = 15.525, *P* = 1.090 × 10^−49^) between PA and NA. We noted that because the polarity of NA scores was reversed, both higher PA and NA scores on the SUBI scale are indicative of a better state of well-being. Table 2 and Figure 1 show the distributions of SUBI scores. Figure 2 shows the scatter plot of the association between PA and NA.

**Table 1.** Demographic variables of the study participants

		Males		Females		
		Mean	Range	Mean	Range	*P*
Age		20.81	18–27	20.59	18–27	0.272
SUBI^a^	PA^b^	37.44	18–52	38.53	22–53	<0.001*
	NA^c^	45.93	24–55	45.26	29–57	0.038*
RAPM^d^		28.79	13–36	28.09	15–36	0.001*
POMS^e^	Tension/anxiety	5.98	0–20	6.62	0–20	0.025^*^
	Depression/dejection	3.54	0–20	4.18	0–20	0.008^*^
	Anger/hostility	2.69	0–18	3.32	0–18	0.001^*^
	Vigor/activity	8.22	0–20	8.01	0–20	0.243
	Fatigue/inertia	6.72	0–20	6.99	0–20	0.537
	Confusion/bewilderment	4.42	0–16	4.85	0–16	0.052

a The WHO Subjective Well-Being Inventory.

b Positive affect.

c Negative affect.

d Raven’s Advanced Progressive Matrix.

e Profile of Mood States.

* p<0.05.

**Table 2.** Distribution of SUBI^a^ scores and association between PA^b^ and NA^c^

	≤18	19–24	25–30	31–36	37–42	43–48	49–54	55–57	Mean	Beta value, *t*-value, *P*-value
PA	1	6	112	354	479	234	23		37.90	(0.410, 15.525, 1.090 × 10^−49^)
NA		1	9	65	238	497	372	27	45.65	

Numbers in the table represent the number of subjects in each score range.

a The WHO Subjective Well-Being Inventory.

b Positive affect.

c Negative affect.

No significant correlation was observed between each SUBI score and RAPM score, whereas there were significant correlations between SUBI scores and POMS subscale scores. The results of the association between SUBI and RAPM and that between SUBI and POMS subscales are shown in Table 3.

**Table 3.** Associations between SUBI^a^ score and psychological variables (RAPM^b^ and POMS^c^)

		PA^d^				NA^e^			
		β	*t*	*P* (uncorrected)	*P* (FDR^f^)	β	*t*	*P* (uncorrected)	*P* (FDR)
RAPM		−0.029	−0.996	0.319	0.319	0.016	0.546	0.585	0.585
POMS	Tension/anxiety	−0.220	−7.865	8.181 × 10^−15^	1.909 × 10^−14^*	−0.374	−14.076	8.981 × 10^−42^	3.143 × 10^−41^*
	Depression/dejection	−0.271	−9.804	6.955 × 10^−22^	2.434 × 10^−21^*	−0.466	−18.349	1.643 × 10^−66^	1.150 × 10^−65^*
	Anger/hostility	−0.187	−6.625	5.206 × 10^−11^	9.111 × 10^−11^*	−0.350	−12.998	3.036 × 10^−36^	5.313 × 10^−36^*
	Vigor/activity	0.351	12.972	4.365 × 10^−36^	3.055 × 10^−35^*	0.168	5.911	4.445 × 10^−9^	5.185 × 10^−9^*
	Fatigue/inertia	−0.179	−6.302	4.143 × 10^−36^	5.801 × 10^−10^*	−0.354	−13.142	6.230 × 10^−37^	1.454 × 10^−36^*
	Confusion/bewilderment	−0.104	−3.636	2.884 × 10^−4^	3.365 × 10^−4^*	−0.324	−11.881	7.609 × 10^−31^	1.065 × 10^−30^*

* *P* < 0.05, corrected for multiple comparisons using false discovery rate.

a The WHO Subjective Well-Being Inventory.

b Raven’s Advanced Progressive Matrix.

c Profile of Mood States.

d Positive affect.

e Negative affect.

f False discovery rate.

### MRI data

#### Whole brain analyses of the correlations between positive and negative aspects of SUBI and MD.

**Fig. 3.**
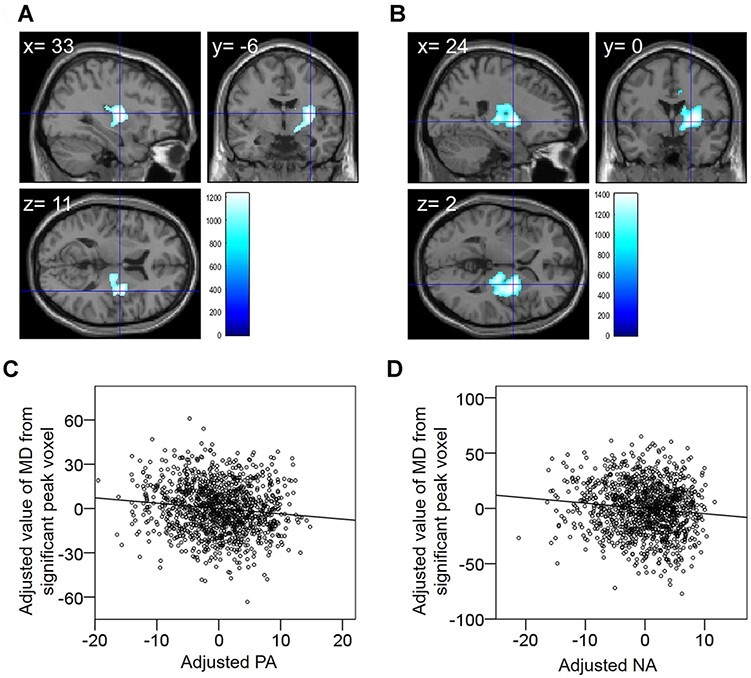
Regions with significant negative correlations between MD and SUBI scores. Results shown were obtained using threshold-free cluster enhancement (TFCE) based on 5000 permutations (*P* < 0.05). Regions with significant correlations were overlaid on a ‘single subject’ T1 image using SPM8. The color represents the strength of the TFCE value. Regions with significant negative correlations between MD and SUBI-PA scores (A). Regions with significant negative correlations between MD and SUBI-NA scores (B). Residual plots are shown with trend lines depicting the correlations between residuals in each multiple regression analysis using the MD value of each significant peak voxel as a dependent variable and PA (C) or NA (D) scores and other confounding factors as independent variables.

After controlling for age, sex, TIV and RAPM score, the whole brain multiple regression analysis showed that PA scores were significantly negatively correlated with MD in the anatomical cluster surrounding the right putamen, insula, pallidus, thalamus, caudate and adjacent areas of WM (Figure 3, Table 4). NA scores were significantly negatively correlated with MD in the anatomical cluster surrounding these same brain regions as well as the middle cingulate gyrus (Figure 3, Table 4). The areas related to the PA and NA of the SUBI overlapped; however, the area related to the NA was broader than that related to the PA. We noted that both higher PA and NA scores on the SUBI scale indicate a better state of well-being. These analyses did not consider the effects of regional GMC, WMC and CSFC differences on MD because non-parametric statistical tests were used that did not consider these effects. However, we carefully removed the CSF in the preprocessing stages and additionally performed region of interest (ROI) analyses to consider these effects; the results did not change the conclusions reached. Detailed descriptions of these ROI analyses are presented in the Supplementary material.

**Table 4.** Brain regions with significant correlation between MD^a^ and the SUBI^b^ scores

	Gray matter areas included	Large bundles included	*x*	*y*	*z*	TFCE^c^ value	Corrected *P*-value (FWE^d^-corrected, TFCE)	Cluster size (voxel)
Negative correlation with PA^e^ score	Right putamen/insula/pallidum/thalamus/caudate body	Right anterior limb of internal capsule/posterior limb of internal capsule/superior corona radiata/posterior corona radiata/external capsule	33	−6	11	1232.09	0.026	1958
Negative correlation with NA^f^ score	Right putamen/thalamus/insula/pallidum/caudate body	Right posterior limb of internal capsule/posterior corona radiata/external capsule	24	0	2	1404.76	0.013	6354
	Right middle cingulate gyrus	Right cingulum	14	−29	32	980.93	0.044	254

a Mean diffusivity.

b The WHO Subjective Well-Being Inventory.

c Threshold-free cluster enhancement.

d Family-wise error.

e Positive affect.

f Negative affect.

#### Whole brain analyses of the correlations between PA and NA of SUBI and FA.

**Fig. 4.**
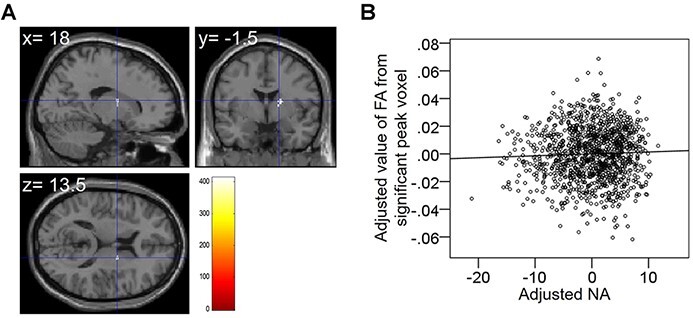
A region with significant negative correlation between FA and SUBI-NA scores. The result shown was obtained using threshold-free cluster enhancement (TFCE) based on 5000 permutations (*P* < 0.05). Regions with significant correlation were overlaid on a ‘single subject’ T1 image using SPM8. The color represents the strength of the TFCE value (A). A residual plot is shown with a trend line depicting the correlation between residuals in a multiple regression analysis using the FA value of each significant peak voxel as a dependent variable and the NA score and other confounding factors as independent variables (B).

After controlling for age, sex, TIV and RAPM scores, the whole brain multiple regression analysis showed that NA scores were significantly positively correlated with FA in the anatomical cluster including the anterior internal capsule (Figure 4, Table 5). No significant correlations were observed between PA scores and FA.

**Table 5.** Brain regions with significant correlation between FA^a^ and SUBI^b^ scores

	Large bundles included	*x*	*y*	*z*	TFCE^c^ value	Corrected *P*-value (FWE^d^-corrected, TFCE)	Cluster size (voxel)
Negative correlation with NA^e^ score	Right anterior limb of internal capsule	18	−1.5	13.5	412.66	0.044	32

a Fractional anisotropy.

b The WHO Subjective Well-Being Inventory.

c Threshold-free cluster enhancement.

d Family-wise error (FWE).

e Negative affect.

## Discussion

To the best of our knowledge, this is the first study that investigates the relationship between DTI measurements (MD and FA) and SWB. We demonstrated—for the first time—that a higher SUBI-PA score, indicative of a higher level of SWB, was significantly associated with a lower MD of brain areas surrounding the right putamen, globus pallidus, insula, caudate and thalamus. Similarly, a higher SUBI-NA score, indicative of a higher level of SWB, was associated with lower MD of the same areas, as well as the middle cingulate gyrus. Consistent with our hypothesis, the areas in which MD significantly correlated with SWB were associated with the dopaminergic system.

Dopaminergic neurons located in the substantia nigra pars compacta project into the striatum, creating the nigrostriatal dopaminergic system (Ungerstedt, 1971). The nigrostriatal dopaminergic system is primarily associated with motor function, as the degeneration of this pathway is reportedly a primary pathological feature of Parkinson’s disease (Sean *et al.*, 1965). Additionally, accumulating evidence implies the involvement of the nigrostriatal dopaminergic system in reward and motivation, which have long been identified as the roles of the mesolimbic dopaminergic system (Wise, 2009). Recently, dopaminergic dysfunction in schizophrenia was determined to be the greatest within nigrostriatal pathways and that dysfunction appeared fundamental to the mechanisms underlying those symptoms (McCutcheon *et al.*, 2019). The globus pallidus receives a dopaminergic input from the substantia nigra (Lindvall and Björklund, 1979) and is thus an important part of the dopaminergic circuitry. Indeed, dopamine reverses reward insensitivity in patients with apathy following globus pallidus lesions (Adam *et al.*, 2013). Previous studies using autoradiography and positron emission tomography (PET) have shown that the thalamus contains a high density of dopamine D2 receptors (Hall *et al.*, 1996; Farde *et al.*, 1997). The thalamus is a part of the corticobasal ganglia circuitry, which has been considered to comprise anatomically and functionally segregated subcircuits, including motor, cognitive and emotional domains (Alexander *et al.*, 1986), as well as exhibits an integrative function for the domains of the circuitry (Haber and Calzavara, 2009). Previous studies have suggested that DA signaling is involved in key neurochemical mechanisms of the insular, striatal and prefrontal regions that cause individual differences in cost/benefit decision-making (Treadway *et al.*, 2012). The insula is important for motivation and dysfunction and may be linked to motivational deficits in individuals with anhedonia (Namkung *et al.*, 2017). Higher NA scores were associated with lower MD in the middle and posterior cingulate cortex, which commonly plays a role in reward processing (Liu *et al.*, 2011). Accordingly, MD of clusters surrounding all these areas may be associated with SWB via dopaminergic system.

As described above, MD is a measure of the directionally averaged magnitude of water diffusion (Pierpaoli *et al.*, 1996, 2001; Le Bihan *et al.*, 2001). Larger spaces between obstacles such as neurons, glial cells and blood vessels should facilitate water diffusion more freely and cause an increase in MD; by contrast, smaller spaces, such as those that arise when cells or blood vessels increase in size or number or when tissue organization is enhanced, prevent water diffusion, thereby decreasing MD (Sagi *et al.*, 2012; Johansen-Berg *et al.*, 2012). As mentioned in previous studies (Nakagawa *et al.*, 2016, 2017), there is a significant negative correlation between dopamine synthesis capacity and MD in the striatum (Kawaguchi *et al.*, 2014).

MD in areas related to the dopaminergic system is reportedly more sensitive in the detection of neuropathology in the dopaminergic system (Seppi *et al.*, 2004; Péran *et al.*, 2010). Compared with healthy individuals, patients with Parkinson’s disease displayed higher MD values in the thalamus, striatum and posterior substantia nigra (Péran *et al.*, 2010; Arribarat *et al.*, 2019). Patients with Parkinson’s disease exhibit a range of neuropsychiatric symptoms, including depression, anxiety, apathy, fatigue, and psychotic symptoms, as well as the loss of motor control (Aarsland *et al.*, 1999, 2009). The impairment of dopaminergic system might be associated with higher MD values and affective disorders. Additionally, this can support the possibility of an association of the dopaminergic system with the negative correlation between MD and SWB observed in the present study.

Previous studies of healthy participants from our research group have revealed an association between MD of subcortical areas and individual traits and states (for review, see Takeuchi and Kawashima, 2018). It was demonstrated that only the motivational state (V/A) was negatively correlated with MD in the right-sided areas, including the globus pallidus, putamen, posterior insula, caudate body and thalamus, whereas the other mood subscales of POMS showed no significant relationships with MD in the whole brain analyses (Takeuchi *et al.*, 2017). The present study results showed that both PA and NA were negatively correlated with MD in these overlapping areas. Additionally, MD in the globus pallidus was found to be associated with some cognitive functions (verbal creativity measured by divergent thinking) and multiple personalities in the Temperament and Character Inventory; the motivational factor may be the key component linking these associations (Takeuchi *et al.*, 2015). Degrees of fatigue were associated with higher MD values in the basal ganglia (Nakagawa *et al.*, 2016), and disruption of the dopaminergic system was proposed as a common mechanism underlying fatigue (Lorist *et al.*, 2009). Furthermore, it has been suggested that motivation is involved in this association (Nakagawa *et al.*, 2016). In reviewing previous findings, decreased MD values in areas highlighted in the present study appear to be associated with a facilitated motivational state of dopaminergic function in healthy participants (Takeuchi and Kawashima, 2018). The present behavioral results that both PA and NA scores were significantly correlated with V/A score from POMS might support this notion, although there were significant correlations between SUBI scores and of the remaining subscales of POMS. Additional thorough investigations are required in the future to identify underlying neural mechanisms of those correlations.

In addition, a higher SUBI-NA score, which was consistent with a higher level of SWB, was associated with higher FA of the right anterior internal capsule. The anterior internal capsule is adjacent to the striatum and contains fibers connecting the subcortical nuclei and frontal cortex (Alexander *et al.*, 1986, 1991; Axer and Keyserlingk, 2000). Fronto–thalamic–striatal connectivity is important for the appreciation of reward, emotional processing and mood state (Sussmann *et al.*, 2009). Moreover, it has been suggested that disturbed frontosubcortical connectivity is a key factor in psychopathology (Mega and Cummings, 1994). Decreased integrity in the structural connections of the frontal lobe and subcortical structures may be associated with lower SWB.

In the present study, the right-sided areas, including the basal ganglia, thalamus and insula specifically, showed a negative correlation with SWB. This is consistent with the right hemisphere hypothesis, which assumes a general dominance of the right hemisphere for all emotions, regardless of affective valence (Ross, 1984; Borod *et al.*, 1998). Moreover, some previous studies investigating the laterality of the dopaminergic dysfunction in Parkinson’s disease suggested that the right basal ganglia plays a greater role in affective processes than the left (Péron *et al.*, 2017; Stirnimann *et al.*, 2018). Based on these findings, the right-sided areas observed in the present study may be associated with a greater role in emotional processing. Numerous previous studies have focused on the association between approach/avoidance motivation and hemispheric asymmetry; therefore, the left hemisphere may play a role in approach-related affect, whereas the right hemisphere may play a role in avoidance-related affect (Sutton and Davidson, 1997; Harmon-Jones *et al.*, 2010). Recently, this asymmetric pattern related to approach/avoidance was identified in the striatal dopamine function (Porat *et al.*, 2014; Aberg *et al.*, 2015). Considering that the result of the present study indicated SWB can be positively associated with the right striatal dopaminergic function, individuals with lower SWB may have poorer capabilities to avoid emotionally negative stimuli.

Significant differences between males and females were observed in SUBI-PA and SUBI-NA scores. Females showed significantly higher PA scores, which were associated with better states of SWB; conversely, males exhibited significantly higher NA scores, which were associated with lesser subjective ill-being states (as well as indicating better states of SWB). The mechanisms underlying these sex differences in SWB remain unclear. Previous research regarding SWB has not provided consistent evidence concerning sex differences (see Batz-Barbarich and Tay, 2018). Moreover, it appears that sex differences in SWB are often small, not universal and depend on the cultural values and societal conditions (Diener *et al.*, 2018a). However, based on the components of PA and NA, previous large-scale, nationally representative studies have provided some findings regarding sex differences. Younger females tended to have higher levels of happiness than younger males, whereas older females have lower levels of happiness than older males (Inglehart, 2002; Easterlin, 2003). A large-scale research study using data from the Gallup World Poll showed that females exhibited higher levels of negative emotion (lower levels of SWB) than males (Zuckerman *et al.*, 2017). Behavioral data in the present study appear to be consistent with these previous findings. Moreover, the comparison of behavioral data in the present study was consistent with the results of a previous study involving an older study population (1618 Japanese participants; age range 20–64 years; male mean age: 46.4 years, female mean age: 46.9 years); the study showed that males had significantly higher PA and NA SUBI scores (Ono and Yoshimura, 2010).

The current study has several limitations that should be noted. First, because this study used a cross-sectional design, the results cannot determine a causal relationship between SWB and MD. To overcome this limitation, a prospective study that confirms such causality is required. Second, the study included young healthy participants who possessed high educational backgrounds. Because previous reviews have suggested that several life circumstances, including, marriage, widowhood, unemployment and disability, affected SWB (Larsen and Eid, 2008; Diener *et al.*, 2018b), our findings cannot be generalized for the entire human population. A larger and more representative sample set is required for such generalization. Third, dopamine was not measured in the present study; thus, future investigations should include more sensitive measures of dopamine function such as PET scans. It should be noted that there is a possibility that neural changes other than those of dopaminergic neurons may affect MD in areas related to the dopaminergic system. For example, previous studies investigating patients with Huntington’s disease, which is characterized by the degeneration of cholinergic and GABAergic neurons in the striatum, found that while dopaminergic neurons are relatively unaffected, MD was increased in areas around the striatum compared with healthy control (Douaud *et al.*, 2009; Sánchez-Castañeda *et al.*, 2013).

In conclusion, the present study is the first to reveal the association between SWB and microstructural properties using DTI. The results showed that individual measurements of SWB are reflected in the variability of the microstructural properties of brain areas involved in the dopaminergic function. Our findings integrate the psychological and physiological aspects of SWB using neuroimaging techniques and advance our understanding of the contribution of our brains to SWB. Our results suggest the possibility of enhancing well-being via physiological changes in the brain.

## Supplementary Material

nsab063_Supp
