# Supplementary material for: Brain microstructural properties related to subjective well-being: diffusion tensor imaging analysis
Source: Soc Cogn Affect Neurosci. 2021 May 14;16(10):1079–90. doi: 10.1093/scan/nsab063 (PMC8483277; doi:10.1093/scan/nsab063)
Supplement: nsab063_Supp [file nsab063_supp.zip › Supplementary methods.docx]

# Supplementary methods

## Preprocessing of imaging data

### Segmentation of structural data

We adopted the two-step segmentation process for segmentation of diffusion images. First, fractional anisotropy (FA) images of each individual were segmented into six tissues using the new segmentation algorithm implemented in SPM8. Default parameters and tissue probability maps were used, except that affine regularization was performed using the International Consortium for Brain Mapping (ICBM) template for east Asian brains, and the sampling distance (the approximate distance between sampled points when estimating the model parameters) was 2 mm. Next, we synthesized the FA images and MD maps of each individual by following the procedures to create new images in which some parts were FA images and other parts were MD maps. In these synthesized images, the areas with a WM tissue probability of > 0.5 in the abovementioned first segmentation process was the FA image multiplied by −1; hence, this synthesized image shows a very clear contrast between the WM and other tissues, the remaining area was the MD map. The synthesized image from each individual was then segmented using the new segmentation with the same parameters as above. We adopted this two-step segmentation process due to the need to combine contrast information between WM and GM from FA images with the MD map, which alone does not show a clear contrast between WM and GM. For more details regarding this two-step segmentation process, see our previous study (Takeuchi *et al.*, 2013).

### Normalization of structural data

After segmentation of the diffusion images, we proceeded with the diffeomorphic anatomical registration through exponentiated lie algebra (DARTEL) registration process implemented in SPM8. In this process, the GM input for the DARTEL process was the DARTEL-imported image of the GM tissue probability map produced in the second new segmentation process. The WM input for the DARTEL process was created as follows. First, the raw FA images were multiplied by the WM tissue probability map produced in the second new segmentation process within the areas with a WM probability of > 0.5 (the signals from other areas were set to 0). The FA multiplied by the WM tissue probability map was used in the DARTEL procedures because this image includes different signal intensities within the WM tissues and improves accuracy of adjusting the image to the template from the perspective of not only the outer edge of the tissue, but also within WM tissues. Then, this FA multiplied by the WM tissue probability map was co-registered and resliced to the DARTEL-imported WM tissue probability map produced in the second new segmentation. The template for the DARTEL procedures was created using imaging data from 63 participants, who had been involved in a previous study (Takeuchi *et al*, 2011), and were also included in the present study. We used a template created from only a portion of participants as n = 63 is enough to create a template and thus cannot be considered to be problematic. Using this existing template, DARTEL procedures were performed on all of the subjects. In these DARTEL procedures, parameters were changed as follows to improve accuracy. The number of Gauss-Newton iterations to be performed within each outer iteration was set to 10 and, for each outer iteration, we used 8-fold more time points than the default to solve the partial differential equations. The number of cycles used by the full multi-grid matrix solver was set to 8. The number of relaxation iterations performed in each multi-grid cycle was set to 8. After running DARTEL, the resulting synthesized images were spatially normalized to the MNI space. Using the parameters for these procedures, the raw MD map, raw FA map, GM segmentation map [GM concentration (density or segmentation) (GMC) map], WM segmentation map [WM concentration (density or segmentation) (WMC) map], and cerebral spinal fluid (CSF) segmentation map [CSF concentration (density or segmentation) (CSFC) map] from the abovementioned second new segmentation process were normalized to give images of 1.5 × 1.5 × 1.5 mm^3^. Subsequently, from the images of the normalized MD map, GMC map, WMC map, or CSFC map, areas that were not strongly likely to be gray or white matter (defined as “gray matter tissue probability + white matter tissue probability < 0.99”) were removed to exclude the strong effects of CSF on MD. Whereas, from the normalized images of the FA, areas that were not strongly to be white matter (defined as “white matter tissue probability < 0.99”) were removed. In these procedures, our custom template created from average images of the normalized GMC and the normalized WMC images of the abovementioned 63 participants were used. Then, MD images were smoothed by convolving them with an isotropic Gaussian kernel of 8-mm full width at half maximum (FWHM), and FA images with 6-mm FWHM. For more details regarding these normalization procedures, see our previous study (Takeuchi *et al.*, 2013).

## ROI analyses

We employed three kinds of regions of interest (ROI) analyses in this study: ROI analysis of areas highlighted by whole brain statistical analyses, ROI analysis for left-sided areas symmetric to those observed in the results of whole brain statistical analysis, and ROI analysis for areas chosen based on *a priori* hypothesis that SWB might be related to MD in regions associated with the dopaminergic system. In each analysis, results with a threshold *p*-value of <0.05 were considered statistically significant after correcting for the FDR using the classical one-stage method (Benjamini and Hochberg, 2000). For the last ROI analysis, we chose areas typically related to the dopaminergic pathways without significant correlation in the whole brain statistical analyses.

### ROI analyses of the associations between MD and SUBI

After identifying the MD correlates of the SUBI, an ROI approach was employed to areas highlighted by the whole brain statistical analyses. As previously reported (Takeuc*hi et al*, 2017), such an ROI analysis can be used to determine whether areas related to the SUBI scores were affected by the extent of MD or GM, WM, and CSF. The areas included the right globus pallidus, right putamen, right caudate body, and right thalamus. All ROIs were constructed using the WFU PickAtlas Tool (<http://fmri.wfubmc.edu/software/pickatlas>) (Maldjian et al., 2003; Maldjian et al., 2004). The mask images of the ROIs, except for the right thalamus, were generated using the Brodmann area option in the PickAtlas tool, whereas those of the right thalamus were generated using the Talairach Daemon option. These mask images were resliced, the defining space for which was a normalized image from a patient. Subsequently, the mean MD, GMC, WMC, and CSFC values of these images were extracted from the normalized and unsmoothed images. To extract these values, we limited the areas to those that showed “gray matter tissue probability + white matter tissue probability >0.999” in the custom template mentioned above. ROIs after abovementioned processes are shown in Supplementary Figure 1a-d. The associations were tested using multiple regression analysis. The dependent variable was the mean MD in one of the ROIs, and the independent variables comprised PA and NA scores, age, sex, and the mean GMC, WMC, and CSFC values of the ROI.

### ROI analyses for the left-sided areas symmetric to those observed in the results of whole brain statistical analyses

We employed ROI approaches to left-sided areas symmetric to those included in the whole brain analyses to confirm the presence of left-right laterality effect of the MD in the ROIs in terms of the association with SUBI. The mask images of the ROIs were generated, and resliced, and the mean MD, GMC, WMC, and CSFC values of these mask images were extracted. Multiple regression analyses were conducted in the same manner described in the abovementioned ROI analyses.

### ROI analyses of the correlations between SUBI and MD in the dopaminergic areas without significant correlation in the whole brain analyses

We employed ROI approaches for areas that are typically associated with the dopaminergic system and do not exhibit significant correlation with SUBI scores in the whole brain statistical analyses. The bilateral nucleus accumbens (NAcc), substantia nigra (SN), and the ventral tegmental area (VTA), which are associated with the dopaminergic system (Wise, 2004), were included. All ROIs were constructed using the WFU PickAtlas tool (<http://fmri.wfubmc.edu/software/pickatlas>) (Maldjian et al., 2003; Maldjian et al., 2004). The mask images of ROIs for the left and right SN were constructed using the Brodmann area option in the PickAtlas tool. The mask images for the left and right NAcc were constructed using the IBASPM71 option considering that ROIs for the NAcc cannot be selected in the Brodmann area options. Furthermore, the mask image of the VTA was set to a 9 × 9 × 9 mm^3^ voxel with the center of x, y, z = 0, − 15, − 12, respectively (Tomasi and Volkow, 2014) because there were no options that included the established area. Then, we constructed the masks of the five ROIs. These mask images of ROIs were resliced and the defining during this procedure was a normalized image from a patient. Subsequently, the mean MD, GMC, WMC, and CSFC values of these mask images were extracted from the normalized and unsmoothed images following the abovementioned manner for ROI analyses. ROIs after abovementioned processes are shown in Supplementary Figure 1e-g. The associations were tested using multiple regression analyses. The dependent variable was the mean MD in one of the ROIs and the independent variables comprised SUBI-PA or SUBI-NA scores, age, sex, and the mean GMC, WMC, and CSFC values of each ROI.

## Analyses of sex differences in the neural correlates of SUBI scores

To investigate the MD (or FA) correlates of SUBI scores between sexes, we used voxel-wise analysis of covariance (ANCOVA) with sex difference as a grouping factor (using the full factorial option of SPM8). ANCOVA for PA and NA were separately performed similarly to the multiple regression analyses described in the main text. In these analyses, age, RAPM score, TIV, and PA or NA scores were included as covariates. The covariates of age, RAPM score, and the PA or NA score were modeled to ensure each covariate had a unique relationship with MD (or FA) for each sex (using the interactions option in SPM8), thereby enabling an investigation of the effects of interaction between sex and each covariate. Correction for multiple comparisons was performed using the same method used in the whole-brain multiple regression analyses.

# Supplementary Results

## ROI analyses for the areas highlighted by the whole brain statistical analyses

In accordance with the results of the whole brain analyses, the areas correlated with the SUBI scores included parts of the right putamen, globus pallidus, caudate body, and thalamus. We performed ROI analyses to investigate potential correlations between the MD of these areas and the SUBI-PA or SUBI-NA scores after correcting for confounding variables of the MRI data (GMC, WMC, and CSFC), age and sex. As shown in Supplementary Table 1, for each SUBI score, there were significant negative correlations between SUBI scores and MD in the right putamen, right globus pallidus, right caudate body, and right thalamus

## ROI analyses for the left-sided areas symmetric to those observed in the whole brain statistical analyses

We performed ROI analyses for the left-sided areas symmetric to those included in the whole brain analyses. As shown in Supplementary Table 2, no significant correlations were found between SUBI scores and MD in the ROIs except for that between PA and MD in the left caudate body.

## ROI analyses of the correlations between SUBI and MD in dopaminergic areas not significantly correlated in whole brain analyses

We performed ROI analyses to investigate whether there was any correlation between MD in areas related to the dopaminergic system that did not exhibit significant correlation in the whole brain statistical analyses and SUBI-PA or SUBI-NA score after correcting for confounding variables of age, sex, TIV, and RAPM score. As shown in Supplementary Table 3, no significant correlation was noted between SUBI scores and MD in any ROIs).

## Sex differences in the neural correlates of SUBI scores

We found no significant effect of interaction between sex and PA or NA score on MD. Significant interactions between NA and sex on FA moderated by the contrasts of positive associations in males and negative associations in females were observed in the left frontal sub-gyral region (Supplementary Table 4, Supplementary Figure 2). No significant effect of interaction between sex and PA score on FA were observed.

# Supplementary Discussion

## ROI analyses of the correlations between SUBI and MD in the dopaminergic areas not significantly correlated in whole brain analyses

Supplementary ROI analyses showed that there were no significant correlations between SUBI scores and MD in the VTA, bilateral NAcc, and SN, which could be considered to be typical dopaminergic areas. It is not yet understood why areas extending to the right putamen, globus pallidus, caudate, thalamus and insula in the dopaminergic system have particularly significant correlations in the SUBI. As mentioned in a previous review, there are possibilities that the smallness of the partial-volume effects of CSF or accumulation of iron can affect MD in these areas (Takeuchi and Kawashima, 2018).

## Sex differences in the neural correlates of SUBI scores

There were significant effects of interaction between sex and NA on FA in the left frontal sub-gyral white matter, which connects the cortical and subcortical areas and is involved in various cognitive and emotional processes (Moretti and Signori, 2016). The integrity of this connection may be related to the behavioral results considering that males had significantly better states of SWB with respect to NA. However, because the mechanism and significance of this association is unclear, future investigations are warranted.

# Supplementary references

Benjamini, Y., Hochberg, Y. (2000). On the Adaptive Control of the False Discovery Rate in Multiple Testing With Independent Statistics. *Journal of Educational and Behavioral Statistics*, **25**, 60–83

Maldjian, J.A., Laurienti, P.J., Burdette, J.H. (2004). Precentral gyrus discrepancy in electronic versions of the Talairach atlas. *NeuroImage*, **21**, 450–55

Maldjian, J.A., Laurienti, P.J., Kraft, R.A., et al. (2003). An automated method for neuroanatomic and cytoarchitectonic atlas-based interrogation of fMRI data sets. *NeuroImage*, **19**, 1233–39

Moretti, R., Signori, R. (2016). Neural correlates for apathy: Frontal-prefrontal and parietal cortical- subcortical circuits. *Frontiers in Aging Neuroscience*, **8**

Takeuchi, H., Kawashima, R. (2018). Mean Diffusivity in the Dopaminergic System and Neural Differences Related to Dopaminergic System. *Current Neuropharmacology*, **16**, 460–74

Takeuchi, H., Taki, Y., Hashizume, H., et al. (2011). Cerebral blood flow during rest associates with general intelligence and creativity. *PLoS ONE*, **6**, 4–12

Takeuchi, H., Taki, Y., Sekiguchi, A., et al. (2017). Mean diffusivity of basal ganglia and thalamus specifically associated with motivational states among mood states. *Brain Structure and Function*, **222**, 1027–37

Takeuchi, H., Taki, Y., Thyreau, B., et al. (2013). White matter structures associated with empathizing and systemizing in young adults. *NeuroImage*, **77**, 222–36

Tomasi, D., Volkow, N.D. (2014). Functional connectivity of substantia nigra and ventral tegmental area: Maturation during adolescence and effects of ADHD. *Cerebral Cortex*, **24**, 935–44

Wise, R.A. (2004). Dopamine, learning and motivation. *Nature Reviews Neuroscience*, **5**, 483–94

**Supplementary Table 1. Matrix of statistical results (beta values, *t*-values, and *p*-values) for the multiple regression analyses of SUBI^a^ scores and MD^b^ in ROIs^c^, with the covariates of age, sex, mean GMC^d^, mean WMC^e^ and mean CSFC^f^ values in each ROI**

|  | **PA^g^** | | | | **NA^h^** | | | |
| --- | --- | --- | --- | --- | --- | --- | --- | --- |
|  | β | *t* | *p* (uncorrected) | *p* (FDR^i^) | β | *t* | *p* (uncorrected) | *p* (FDR) |
| Right putamen | −0.09 | −3.232 | 1.263 × 10^−3^ | 2.921 × 10^−3^* | −0.077 | −2.743 | 6.170 × 10*^−^*^3^ | 2.792 × 10^−3^* |
| Right globus pallidus | −0.089 | −3.19 | 1.461 × 10^−3^ | 2.921 × 10^−3^* | −0.09 | −3.203 | 1.396 × 10^−3^ | 2.792 × 10^−3^* |
| Right caudate body | −0.081 | −2.915 | 3.623 × 10^−3^ | 4.459 × 10^−3^* | −0.075 | −2.696 | 7.112×10^−3^ | 7.112 × 10^−3^* |
| Right thalamus | −0.078 | −2.849 | 4.459 × 10^−3^ | 4.459 × 10^−3^* | −0.092 | −3.329 | 8.969×10^−4^ | 7.112 × 10^−3^* |

**p* < 0.05, corrected for multiple comparisons using FDR

^a^ WHO-subjective well-being inventory

^b^ Mean diffusivity

^c^ Regions of interest

^d^ Gray matter concentration

^e^ White matter concentration

^f^ Cerebrospinal fluid concentration

^g^ Positive affect

^h^ Negative affect

^i^ False discovery rate

# **Supplementary Table 2.** Matrix of statistical results (beta values, *t*-values, *p*-values) for the multiple regression analyses of SUBI^a^ scores and MD^b^ in ROI^c^, for the left-sided areas symmetric to those observed in the results of whole brain statistical analyses, with the covariates of age, sex, mean GMC^d^, mean WMC^e^, and mean CSFC^f^ values in each ROI

|  | **PA^g^** | | | | **NA^h^** | | | |
| --- | --- | --- | --- | --- | --- | --- | --- | --- |
|  | β | *t* | *p* (uncorrected) | *p* (FDR^i^) | β | *t* | *p* (uncorrected) | *p* (FDR) |
| Left putamen | −0.038 | −1.34 | 0.182 | 0.24 | −0.050 | −1.73 | 0.083 | 0.11 |
| Left globus pallidus | −0.053 | −1.83 | 0.067 | 0.13 | −0.065 | −2.26 | 0.024 | 0.10 |
| Left caudate body | −0.074 | −2.59 | 0.010 | 0.04* | −0.053 | −1.85 | 0.065 | 0.11 |
| Left thalamus | 0.007 | 0.23 | 0.820 | 0.82 | −0.037 | −1.28 | 0.201 | 0.20 |

* *p* < 0.05, corrected for multiple comparisons using FDR

^a^ WHO-subjective well-being inventory

^b^ Mean diffusivity

^c^ Regions of interest

^d^ Gray matter concentration

^e^ White matter concentration

^f^ Cerebrospinal fluid concentration

^g^ Positive affect

^h^ Negative affect

^i^ False discovery rate

# **Supplementary Table 3.** Matrix of statistical results (beta values, *t*-values, *p*-values) for the multiple regression analyses of SUBI^a^ scores and MD^b^ in ROI^c^, with the covariates of age, sex, mean GMC^d^, mean WMC^e^, and mean CSFC^f^ values in each ROI

|  |  | **PA^g^** | | | | **NA**^h^ | | | |
| --- | --- | --- | --- | --- | --- | --- | --- | --- | --- |
|  |  | β | *t* | *p* (uncorrected) | *p* (FDR^i^) | β | *t* | *p* (uncorrected) | *p* (FDR) |
| SN^j^ | L^k^ | −0.041 | −1.418 | 0.156 | 0.164 | −0.033 | −1.136 | 0.256 | 0.336 |
|  | R^l^ | −0.055 | −2.023 | 0.043 | 0.103 | −0.04 | −1.463 | 0.144 | 0.269 |
| VTA^m^ |  | −0.056 | −1.945 | 0.052 | 0.103 | − 0.079 | −2.760 | 0.154 | 0.269 |
| Nacc^n^ | L | −0.054 | −1.893 | 0.059 | 0.103 | − 0.048 | −1.661 | 0.097 | 0.269 |
|  | R | −0.049 | −1.742 | 0.082 | 0.107 | − 0.014 | −0.478 | 0.633 | 0.665 |

^a^ WHO-subjective well-being inventory

^b^ Mean diffusivity

^c^ Regions of interest

^d^ Gray matter concentration

^e^ White matter concentration

^f^ Cerebrospinal fluid concentration

^g^ Positive affect

^h^ Negative affect

^i^ False discovery rate

^j^ Substantia nigra

^k^ Left

^l^ Right

^m^ Ventral tegmental area

^n^ Nucleus accumbens

# Supplementary Table 4. Brain regions exhibiting significant effects of the interaction between SUBI-NA^a^ scores and sex (moderated by positive correlation in males and negative correlation in females) on FA^b^

|  | x | y | z | TFCE^c^ values | Corrected *p*-values (FWE^d^-corrected, TFCE) | Cluster size (voxel) |
| --- | --- | --- | --- | --- | --- | --- |
|  |  |  |  |  |  |  |
| Left hemisphere, sub-gyral white matter | −19.5 | −34.5 | 33 | 418.93 | 0.037 | 55 |

^a^ Negative affect (NA) of the subjective well-being inventory (SUBI)

^b^ Fractional anisotropy

^c^ Threshold-free cluster enhancement

^d^ Family-wise error (FWE)

**Supplementary Figure 1.**


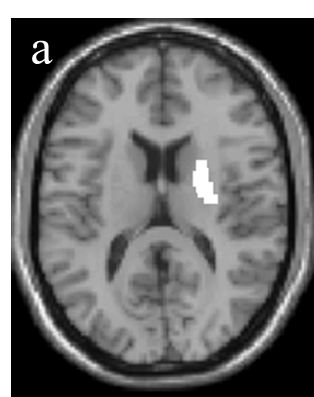

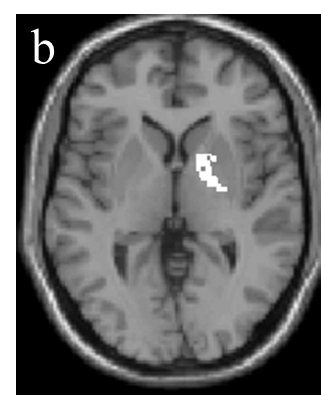


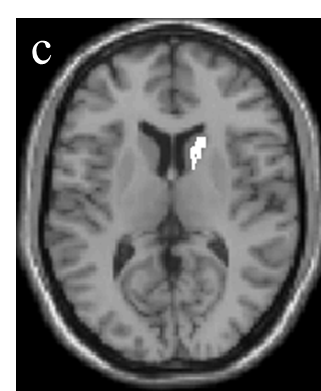

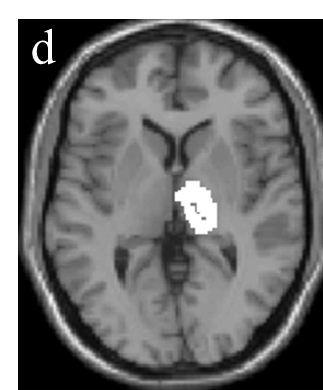


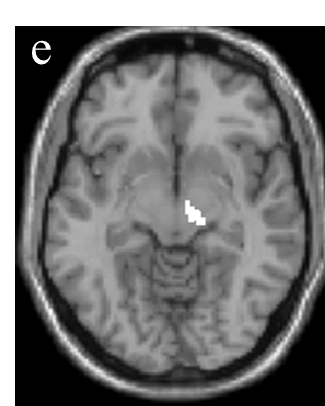

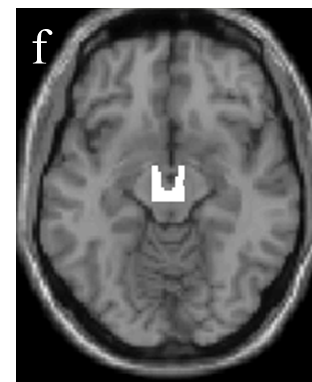

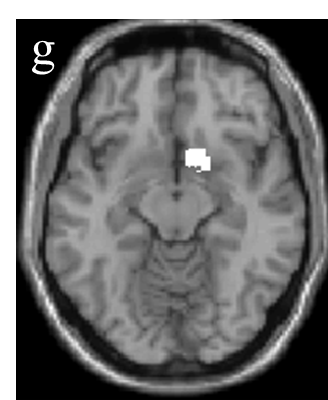


**Supplementary Figure 2.**


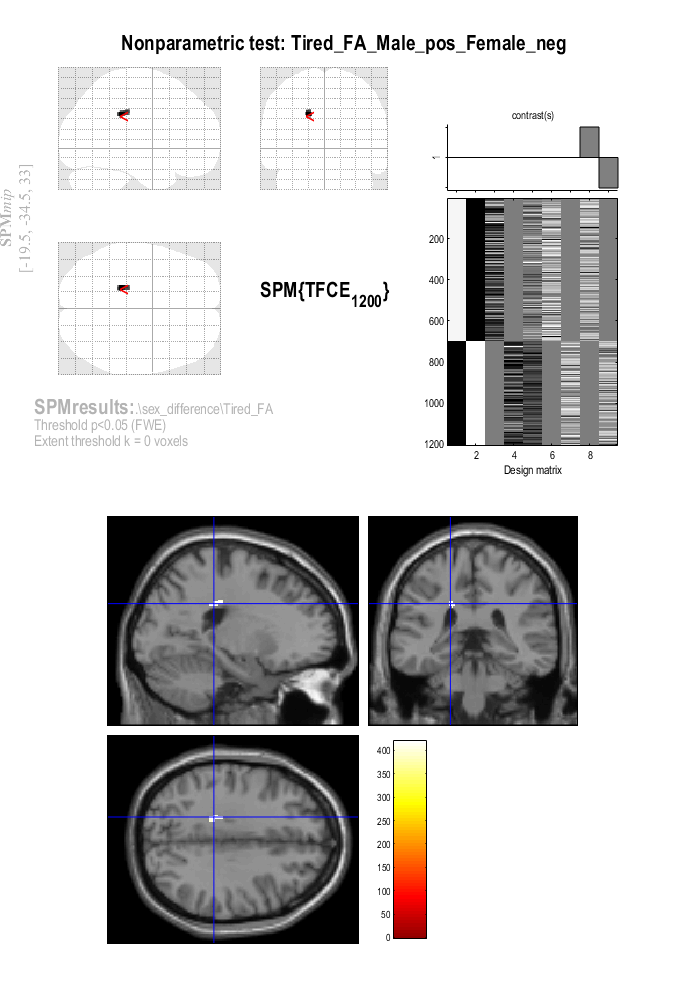


**x= -19.5**

**y= -34.5**

**z= 33**

**Figure legends**

Supplementary Figure 1.

Brain regions exhibiting ROIs created in the supplementary methods and overlaid on a “single subject” T1 image in SPM8. These areas included (a) the right putamen, (b) right globus pallidus, (c) right caudate body, (d) right thalamus, (e) right substantia nigra, (f) ventral tegmental area, and (g) right nucleus accumbens.

Supplementary Figure 2.

Brain regions exhibiting significant effect of interaction between NA scores and sex (that are moderated by positive correlation in males and negative correlation in females) on FA. The result shown was obtained using threshold-free cluster enhancement (TFCE), P < 0.05 based on 5000 permutations. Regions with significant correlations are overlaid on a “single subject” T1 image in SPM8. The color represents the strength of the TFCE value. The significant interactions between NA and sex on FA that were moderated by the contrasts of positive associations in males and negative associations in females were found in the left frontal sub-gyral region.
